# Identification of a novel disulfideptosis-related gene signature for prognostic implication in lower-grade gliomas

**DOI:** 10.18632/aging.205688

**Published:** 2024-03-27

**Authors:** Fuqiang Zhang, Meihong Lv, Yi He

**Affiliations:** 1 Department of Neurosurgery, The Second Affiliated Hospital of Dalian Medical University, Dalian, Liaoning, China; 2 Department of Anesthesiology, The First Affiliated Hospital of Dalian Medical University, Dalian, Liaoning, China; 3 Department of Urology, The Second Affiliated Hospital of Dalian Medical University, Dalian, Liaoning, China

**Keywords:** IQGAP1, GBMLGG, nomogram, clinical prognosis

## Abstract

Lower-grade gliomas (GBMLGG) are common, fatal, and difficult-to-treat cancers. The current treatment choices have impressive efficacy constraints. As a result, the development of effective treatments and the identification of new therapeutic targets are urgent requirements. Disulfide metabolism is the cause of the non-apoptotic programmed cell death known as disulfideptosis, which was only recently discovered. The mRNA expression data and related clinical information of GBMLGG patients downloaded from public databases were used in this study to investigate the prognostic significance of genes involved in disulfideptosis. In the Cancer Genome Atlas (TCGA) and Gene Expression Omnibus (GEO) cohort, our findings showed that many disulfidptosis-related genes were expressed differently in normal and GBMLGG tissues. It was discovered that IQ motif-containing GTPase-activating protein 1 (IQGAP1) is a key gene that influences the outcome of GBMLGG. Besides, a nomogram model was built to foresee the visualization of GBMLGG patients. In addition, *in vivo* and *in vitro* validation of IQGAP1’s cancer-promoting function was done. In conclusion, we discovered a gene signature associated with disulfideptosis that can effectively predict OS in GBMLGG patients. As a result, treating disulfideptosis may be a viable alternative for GBMLGG patients.

## INTRODUCTION

Gliomas are the most prevalent type of primary malignant tumors in the brain [1]. Gliomas are categorized as lower-grade and extremely aggressive high-grade gliomas according to the WHO 2016 grading system. Grade I is primarily benign, while diffuse low-grade and intermediate-grade gliomas comprise WHO grade II and III lesions. Primary glioblastoma multiforme (GBM) and secondary glioblastomas (formed from lower-grade gliomas) make up the grade IV gliomas [2]. Lower-grade gliomas (GBMLGG), including WHO grades II and III, always grow slowly, invade, and sometimes get worse. They account for approximately 1/5 of adult cases of brain cancer [3]. Common molecular markers, like the 1p/19q codeletion status and the isocitrate dehydrogenase (IDH) mutation, can be used to further categorize the majority of GBMLGG. [4]. The IDH1/2 mutation is one of the most important molecular differences in glioma classification. It suggests a pretty good prognosis and a possible therapeutic target. The combined deletion of chromosomes 1p/19q also points to a pretty good outlook and a high level of sensitivity to chemoradiation and alkylating agents. GBMLGG often have better prognoses with malignant aggressivity made in therapy, such as surgical resection and chemotherapy, but higher-grade gliomas have worse prognoses because of their malignant aggressivity [5]. The prognosis is dire for patients with GBMLGG, even though their 5-year overall survival (OS) is 85%; progression-free survival (PFS) for those with untreatable or residual disease that requires treatment is about 40% [6]. Meanwhile, it was found that the prognosis worsens when GBMLGGs advance, with about 70% of individuals experiencing this within ten years [7]. Although grade IV tumors have a worse prognosis than lower-grade gliomas, 70% of patients in the latter group experience high-grade transformation within ten years. Therefore, characterization of distinct and useful molecular markers is urgently needed for the precise diagnosis, tailored treatment, and prognostic evaluation of GBMLGG.

A strong link between cancer and disulfide metabolism has been established in recent years. Disulfide digestion alludes to the redox responses inside cells, where the formation and disruption of disulfide bonds play a crucial role. Numerous cancer cells are subjected to oxidative stress, which results in disulfide metabolism abnormalities and impairs the survival and multiplication of cancer cells, according to recent research [8, 9]. Additionally, biological processes such as immune evasion, metastasis, and drug resistance are linked to disulfide metabolism in cancer cells [10, 11]. Disulfidptosis, a novel type of programmed cell death (PCD), may be linked to the immune response to tumors. Tumour immune cells may be able to recognise the disulfidptosis-induced cell death signal. This will improve the efficacy of cancer treatment by stimulating the immune response of tumor-specific T cells and enhancing the effectiveness of both humoral and cellular immunity. However, additional biomarkers related to disulfide metabolism and connections between targets, disulfide metabolism-dependent pathways, and cancer susceptibility need to be established.

The IQGAP protein family has been around for a long time and is still found in eukaryotes. It includes IQGAP1 (IQ motif-containing GTPase-activating protein 1) [12, 13]. When IQGAP1 was first found as a new sequence in human osteosarcoma tissue in 1994 [14], it was thought to work as a GTPase-activating protein (GAP) that helps signal terminating because of the similarity of its sequence to that of other GAPs. Nonetheless, recent examination indicated that, as opposed to terminate signals, IQGAP1 restrains characteristic GTPase exercises of restricting adjuvants like RAC1 and CDC42, in this manner balancing out the dynamic type of these G proteins [15]. IQGAP1 plays important roles in a variety of biological processes like cell adhesion, invasion and proliferation [10, 16–21], and has been researched as a scaffolding protein for several important oncogenic pathways [22, 23].

Through integrative bioinformatics analyses, our objective is to identify every gene linked to disulfideptosis and understand how it affects prognosis and tumor microenvironment infiltration in GBMLGG. Experiments conducted *in vivo* and *in vitro* further supported the findings.

## MATERIALS AND METHODS

### Data collection and procession

**Figure 1:**
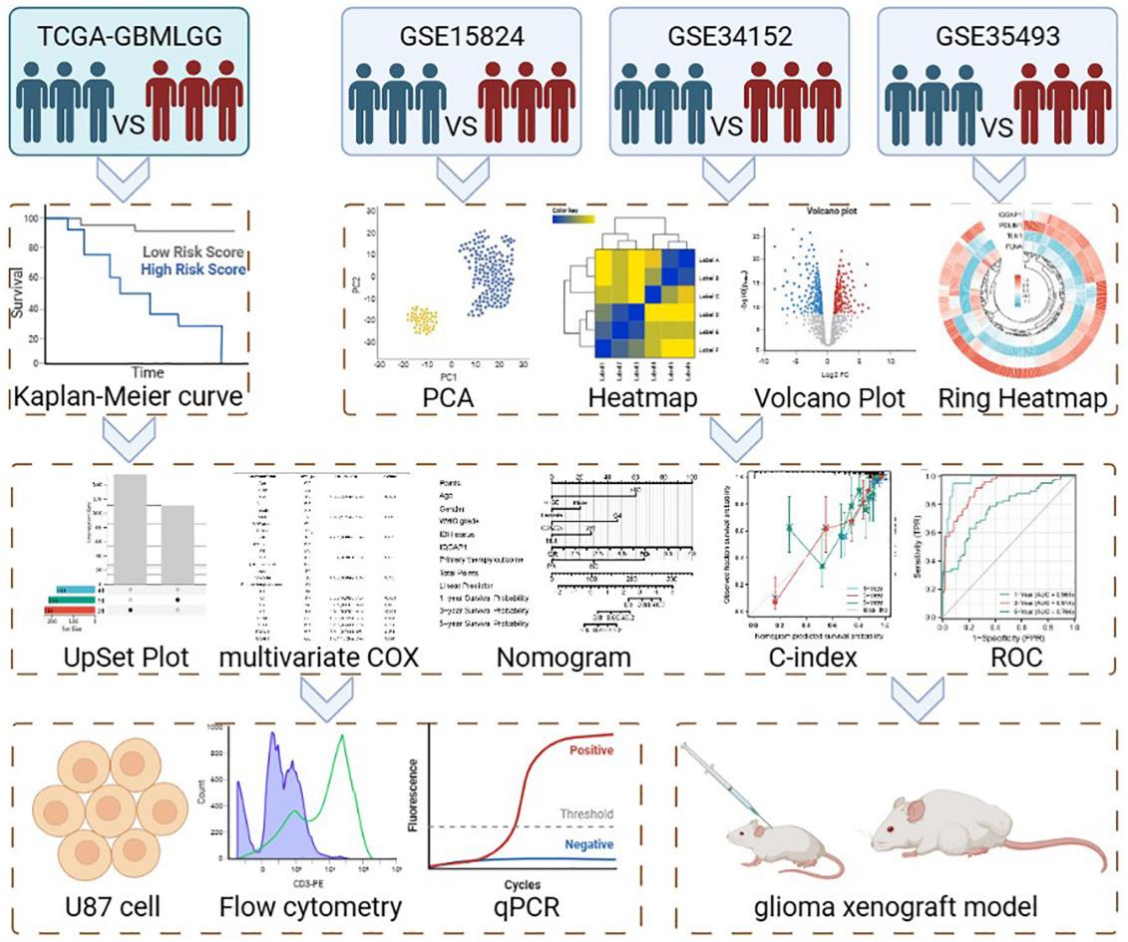
Flow chart of this study.

As shown in Figure 1, 696 GBMLGG patients’ mRNA-sequencing data and associated clinical information were obtained from the Cancer Genome Atlas (TCGA) data set. (https://www.cancer.gov/about-nci/association/ccg/research/underlyinggenomics/tcga, (which was accessed on 22 April 2023)). The specific information about TCGA-GBMLGG is presented in Table 1. What’s more, the microarray information profiles of GSE15824, GSE34152, and GSE50161 in view of a similar stage GPL570 and comparing clinical data were gotten from the Gene Expression Omnibus (GEO) data set (https://www.ncbi.nlm.nih.gov/geo/, (which was accessed on 22 April 2023)). After that, batch effects were removed and the two datasets were combined using the “sva” R package.

**Table 1:** Clinical characteristics of the TCGA-GBMLGG.

**Characteristic**	**Levels**	**Overall**
*n*		696
WHO grade, *n* (%)	G2	224 (35.3%)
	G3	243 (38.3%)
	G4	168 (26.5%)
IDH status, *n* (%)	WT	246 (35.9%)
	Mut	440 (64.1%)
1p/19q codeletion, *n* (%)	Codel	171 (24.8%)
	Non-codel	518 (75.2%)
Gender, *n* (%)	Female	298 (42.8%)
	Male	398 (57.2%)
Age, *n* (%)	≤60	553 (79.5%)
	>60	143 (20.5%)
Race, *n* (%)	Asian	13 (1.9%)
	Black or African American	33 (4.8%)
	White	637 (93.3%)
Histological type, *n* (%)	Astrocytoma	195 (28%)
	Glioblastoma	168 (24.1%)
	Oligoastrocytoma	134 (19.3%)
	Oligodendroglioma	199 (28.6%)
OS event, *n* (%)	Alive	424 (60.9%)
	Dead	272 (39.1%)
Primary therapy outcome, *n* (%)	PD	112 (24.2%)
	SD	147 (31.8%)
	PR	64 (13.9%)
	CR	139 (30.1%)
Age, median (IQR)		45 (34, 59)

### Development and validation of a prognostic disulfidptosis-related gene signature

After downloading the GEO query package from the GEO database, the probes that corresponded to different compounds were eliminated. Only the probes with the highest signal value were kept when multiple probes relating to the same molecule were found. Then the clustering among sample groups was checked through PCA maps, and the difference analysis between the two groups was carried out by the limma package. In the TCGA cohort, we employed Kaplan-Meier analysis to examine the variations in OS between the high-risk and low-risk groups. A significance threshold was set at p < 0.05. The expression of genes linked to disulfidptosis was compared in normal and tumor samples from the GEO cohort using the “limma” R program. When the *p*-value was less than 0.05, genes were deemed to be significantly different. We use the KEGG rest API (https://www.kegg.jp/kegg/rest/keggapi.html) to retrieve the most recent KEGG Pathway gene annotation for the purpose of enriching gene set function analysis, as background, the difference of gene mapping to the background in the collection, the R software package clusterProfiler (version 3.14.3) was used to conduct enrichment analysis and produce the gene set enrichment results. Put five as the minimum gene and five thousand as the maximum gene, a *P*-value of less than 0.05 was deemed statistically noteworthy. For the functional enrichment analysis of gene set, we used the GO annotation of genes in the R software package org.Hs.eg.db (version 3.1.0) as the background to map the differential genes into the background set. The R software package clusterProfiler (version 3.14.3) was used to conduct enrichment analysis and produce the gene set enrichment results. Put five as the minimum gene and five thousand as the maximum gene, a *P*-value of less than 0.05 was deemed statistically noteworthy. For Gene set enrichment analysis (GSEA), we separated the sample into two groups based on glioma and normal tissue, using the GSEA software (version 3.0) from GSEA (https://doi.org/10.1073/pnas. 0506580102, http://software.broadinstitute.org/gsea/index.jsp), and derived Molecular Signatures Database (https://doi.org/10.1093/bioinformatics/btr260, http://www.gsea-msigdb.org/gsea/downloads.jsp) to download the c2. Cp. Kegg. V7.4. Symbols. The GMT Set, to evaluate the relevant pathways and molecular mechanisms, based on gene expression profiles and phenotypic grouping, put five as the minimum gene and five thousand as the maximum gene, 1000 resampling, a *P*-value of less than 0.05 was deemed statistically noteworthy. Utilizing the TCGA and GEO datasets, we distinguished the convergence of qualities with genuinely tremendous contrasts. In addition, a multivariate COX analysis of OS revealed that the aforementioned genes had prognostic values (*p*-values below 0.05).

### Clinical features and establishment of a prognostic nomogram

We used the “rms” R package to make a nomogram for predicting OS based on the clinical information and gene expression profiles of GBMLGG patients in the TCGA cohort. Time-subordinate alignment bends and AUC bends were attracted to check the legitimacy of the nomogram. We further performed both univariate and multivariate Cox regression analyses to investigate whether this prognostic model could on its own predict OS in GBMLGG patients.

### Protein expression analysis

We reviewed cancer Omics data using the University of Alabama Cancer Database (UALCAN) portal [24]. Using the UALCAN portal, protein expression was also looked at in the dataset from the Clinical proteomic tumor analysis consortium (CPTAC). Our examination group assessed the degrees of HNRNPC (NP 001070910.1) all out protein and phosphoprotein articulation in essential cancer and typical tissues subsequent to entering the expression “HNRNPC” into the pursuit box.

### DNA methylation

TCGA-GBMLGG in the Illumina human methylation 450 methylation data and the project level 3 HTSeq - RNAseq FPKM format data. RNAseq information in FPKM design was changed over into TPM design and log2 changed. In this review, the ggplot2 bundle was mostly utilized for perception.

### Patient samples and immunohistochemistry (IHC)

3 paired paraffin-embedded GBMLGG and counterpart normal adjacent tissues were collected from the second hospital of Dalian Medical University. Using IHC, the protein expression levels of IQGAP1 were found in 4-μm tissue slices. Sections were rehydrated in varying alcohols after being deparaffinized in xylene. Sections underwent a 20-minute, 95°C pretreatment in citrate buffer (0.01 mol/L citric acid, pH 6.0). They were then submerged in PBS containing 3% H_2_O_2_ for ten minutes at room temperature. Following treatment with 10% normal goat serum in PBS for 30 minutes at room temperature, the tissue slices were incubated with rabbit polyclonal anti-IQGAP1 antibodies (1:1000 dilution) (ab86064, Abcam) for an entire night at 4°C. Following a PBS washing, the sections were treated with 3,30-diaminobenzidine chromogen for five minutes at room temperature and incubated for twenty minutes with biotinylated goat anti-rabbit IgG. Sections were then counterstained for six minutes using hematoxylin. The second hospital of Dalian Medical University’s Ethics Committee accepted the portion of the study that involved experimentation on human tissues. We acquired informed consent from each and every participant.

### Cell culture and transfections

The American Type Culture Collection (ATCC) provided the human glioma cell lines U87, which were then cultured in DMEM media (Gibco, USA) supplemented with 10% fetal bovine serum (Hyclone, USA), Penicillin-Streptomycin (100 U/ml, Hyclone), and glutamine (2 mM, Hyclone) at 37°C in a humidified environment with 5% CO_2_. After being separated by 0.25% trypsin and 0.02% EDTA solution, the cells were subcultured once every two to three days.

The shRNA targeting IQCAP1 (sh-IQGAP1: 5′-CAACGACATTGCCAGGGATAT-3′) and the control non-silencing RNA (PLV-Ctrl: 5′-GGAATCTCATTCGATGCATAC-3′) were presented by Dong et al. [25]. The shRNA-containing lentiviruses were packaged by co-transfecting the pLVTHM-GFP vector together with psPAX2.0 and pMD2.G into 293T cells. U87 cell suspensions of 5 × 104 cells were cultured in 6-well plates for 24 h at 30% to 40% confluence, and then infected with lentiviruses at 10 multiplicity of infection (MOI), which were estimated by a range of 5, 7.5, 10, 12.5, 15 and 20. After 8 hours, the medium was switched, and if more than 80% of the cells were GFP-fluorescent when seen under an Olympus IX71 fluorescent microscope 72 hours after infection, the cells were screened with puromycin (2 μg/ml). Quantitative real-time PCR analysis verified the transfection effectiveness of IQGAP1.

### Flow cytometry analysis of the cell cycle

Cells were reaped and cultivated in 6-well plates. Cells were digested and collected after 24 hours of incubation, then centrifuged for 5 minutes at 4°C at 1000 rpm. After being washed and suspended in ice-cold PBS, the deposited cells were then resuspended in 70% ethanol for 30 minutes at 4°C. After that, the cells were cleaned and suspended once more for 30 minutes at 4°C in 100 mL of PBS containing the final concentration of 50 mg/mL of RNase A (Sigma, US) and 0.25% Triton X-100. Afterwards, the cells were stained with 10 mg/mL propidium iodide (PI) (Life Technologies, USA) for thirty minutes. After that, a FACScan flow cytometer (Becton Dickinson, USA) was used to immediately analyze the cells. The examples were all examined multiple times, and the small part of every cell cycle stage was estimated.

### Cell apoptosis analysis

Six-well plates were used to seed the cells at a density of 1 × 10^5^ cells per well. There was 2 ml of culture medium in each well. After being incubated for 24 hours, the cells were given 20 milligrams of cisplatin, rinsed with PBS, and then resuspended in 300 milliliters of binding buffer. Subsequently, the cell suspensions were incubated for 15 minutes in complete darkness with 5 μl of PI added. The next step involved using a BD FACSVerse stream cytometer to identify cell apoptosis.

### Quantitative real-time PCR

As directed by the manufacturer, RNA was extracted from U87 cells using the RNA isoPlus^®^ Reagent Kit (Takara Biotechnology, Japan). To convert RNA into cDNA, the PrimeScript^®^ RT Reagent Pack (Takara, Shiga, Japan) was used. Using the SYBR^®^ Premix Ex Taq™ Unit and the 7500 Ongoing PCR Framework (Applied Biosystems, 7500 Continuous PCR Framework, Thermo, USA), the cDNA was enhanced. The conditions for cycling were as follows: The results were analyzed using the comparative Ct technique, with GAPDH acting as the loading control for the target genes during the forty cycles of 30 s at 95°C and 34 s at 60°C. The preliminaries were as per the following: IQGAP1 (Forward: 5′-ACCGTGGACCCAAAGAAC-3′, turn around: 5′-CTTCCCGTAGAACTTTTTGTTG-3′; GAPDH (Following: 5′-GCACCGTCAAGGCTGAGAAC-3′, switch: 5′-TGGTGAAGACGCCAGTGGA-3′).

### Cell proliferation assay

96 well plates were utilized to seed cells (2 × 10^5^ cells/well). The cells were incubated for three hours at 37°C and 5% CO_2_ using the CCK-8 kit from Tiangen (Hangzhou, China), which was mixed at a volume of 10 L per well. At long last, we read the absorbance at 450 nm on the microplate peruser (Thermo Fisher Logical, Inc., USA).

### Experimental glioma xenograft model

Ten male BALB/c nude mice, weighing 20 g and aged 4–6 weeks, were obtained from the Experimental Animal Center of Dalian Medical University (Dalian, China). The animals were kept in housing with a 12-hour light/dark cycle and free access to food and water. The research center circumstances were kept up with at 22°C ± 2°C and an overall dampness of 60% ± 5%. The regulations and guidelines established by the National Institutes of Health were strictly adhered to throughout the execution of each experimental protocol. The Dalian Medical University Experimental Animal Committee (Dalian, China) approved this study.

1 × 10^7^ U87 cells expressing sh-IQGAP1 or sh-Ctrl were implanted subcutaneously in the left axilla after being suspended in 75 μl of DMEM and 75 μl of Matrigel (Collaborative Biomedical Products, USA). The tumor volume was calculated using Super Nova^®^ PET/CT (SNPC-103, Pingsheng Medical Technology (Kunshan Co., Ltd., China) during the 14-day termly observation of the xenograft tumor development.

### Statistical analysis

ANOVA, or one-way analysis of variance, was used to examine group differences. The statistical analyses were performed using the SPSS 25.0 software. All examinations were performed freely and rehashed multiple times. *P* < 0.05 was viewed as genuinely critical.

## RESULTS

### Identification of prognostic disulfidptosis-related DEGs in the TCGA cohort

**Figure 2:**
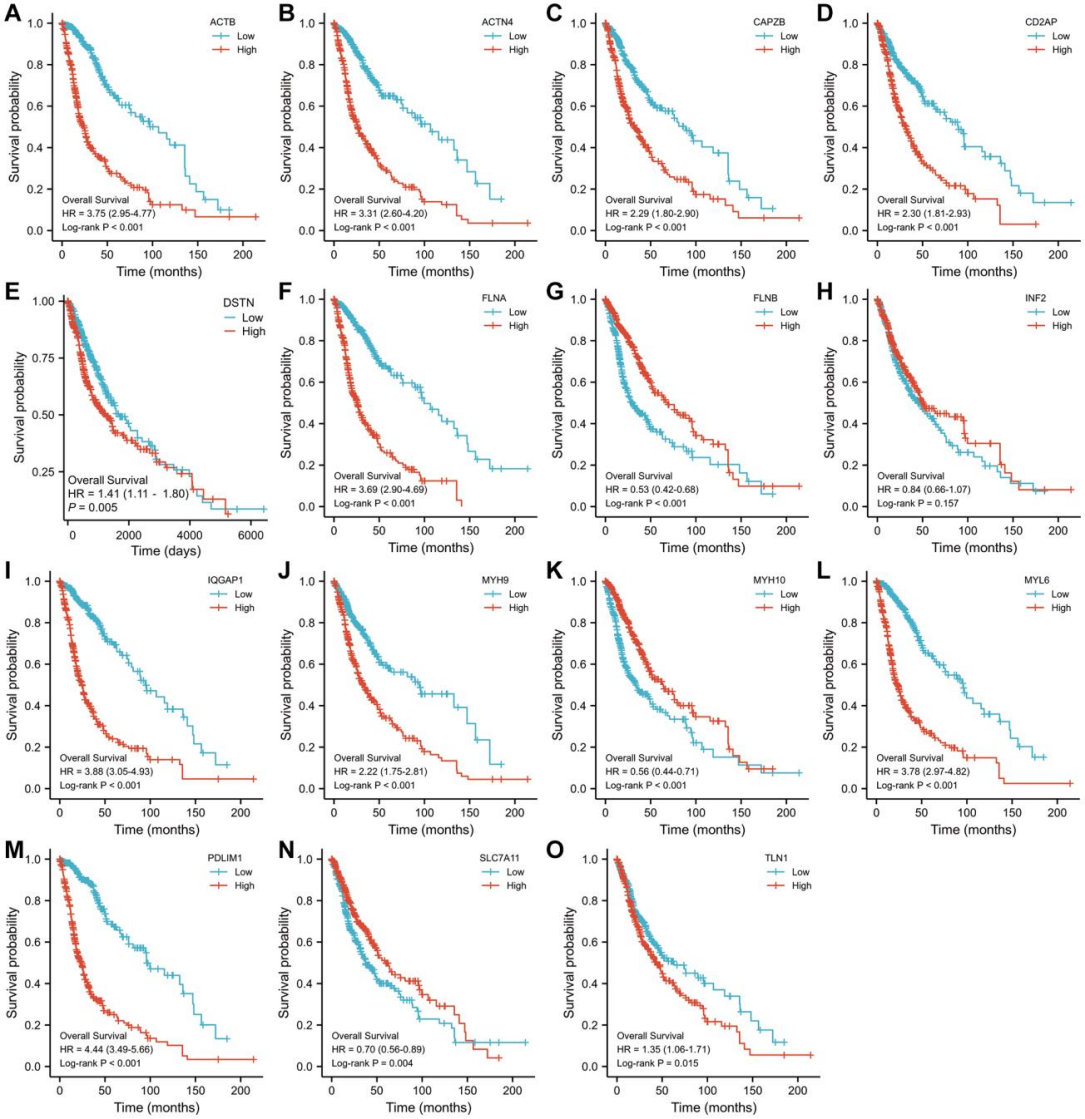
**The Kaplan–Meier OS curves for patients in the high- and low-risk groups in the TCGA cohort (log-rank test).** (**A**) ACTB, (**B**) ACTN4, (**C**) CD2AP, (**D**) CAPZB, (**E**) DSTN, (**F**) FLNA, (**G**) FLNB, (**H**) INF2, (**I**) IQGAP1, (**J**) MYH9, (**K**) MYH10, (**L**) MYL6, (**M**) PDLIM1, (**N**) SLC7A11, (**O**) TLN1.

A log-rank examination uncovered a sum of 14 qualities connected with visualization with a p-value under 0.05 (Figure 2). Among them, a worse prognosis was associated with high expression of 11 genes related to disulfideptosis. Patients with a better prognosis may have higher levels of expression of three genes related to disulfidptosis.

### Identification of differentially expressed disulfidptosis-related genes clusters in the GEO cohort

**Figure 3:**
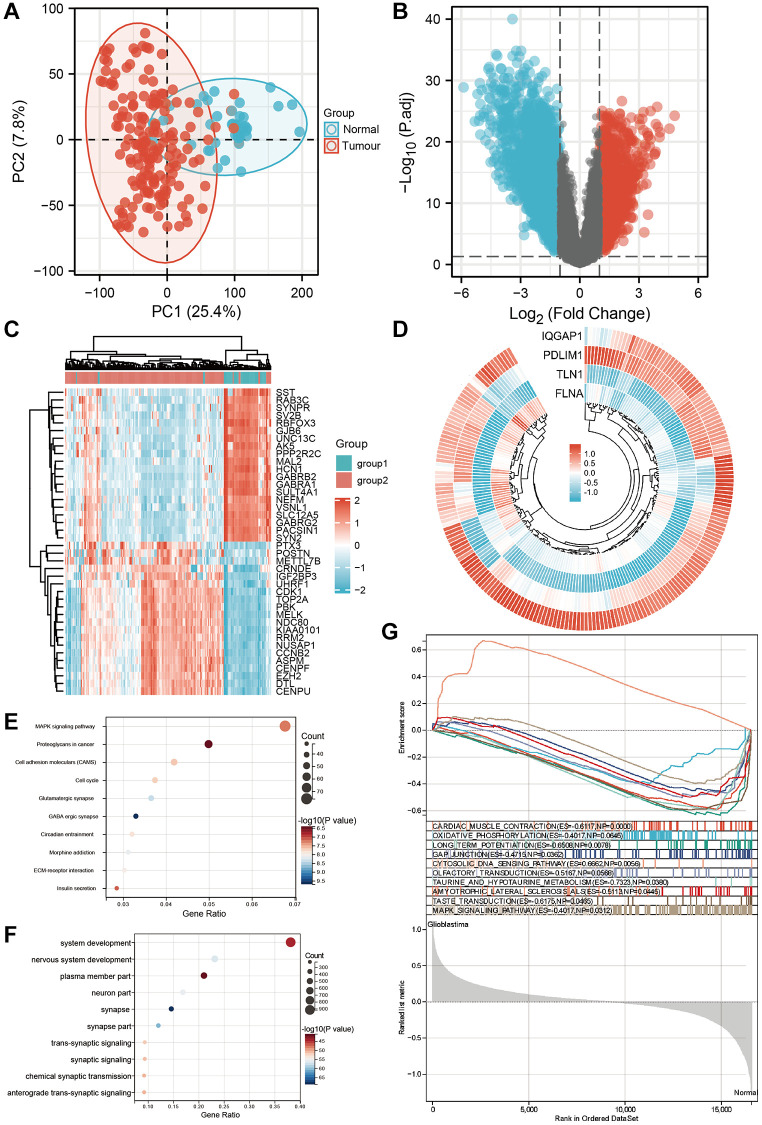
**Screening differential genes in GEO datasets.** (**A**) Principal component analysis showed the clustering between tumor group and normal group of GEO combined dataset. (**B**) The volcano plot shows the differential expression between tumor group and normal group of GEO combined dataset. (**C**) The heat map showed the differential expression between tumor group and normal group of GEO combined data set. (**D**) The ring heat map showed the common differentially expressed genes between tumor group and normal group of TCGA dataset and GEO combined dataset. (**E**) KEGG analysis of differential genes. (**F**) GO analysis of differential genes. (**G**) Gene set enrichment analysis of GEO dataset.

We gathered 209 samples (166 GBMs and 43 normal ones) after combining the GSE15824, GSE34152, and GSE35493 (platform GPL570) datasets. Figure 3A shows that the samples from the two groups are separated in the PCA diagram, demonstrating that the two groups’ differences are clear and that the outcomes of the difference analysis that follows will be more insightful. The criterion for |logFC|>1 and *p*-value 0.05 for genes that are differently expressed in tumor and normal tissue are shown in the volcano figure (Figure 3B). The expression of each of the top 20 genes in the expression profile was visualized using a heat map (Figure 3C). The ring heat map shows the four differentially communicated disulfidptosis-related qualities in the GEO dataset (Figure 3D). In KEGG analysis, the expression of MAPK pathway was the most different (Figure 3E). In the GO analysis, the expression of system development was most different in glioma and normal tissue (Figure 3F). In GSEA, the expression of CARDIAC_MUSCLE_CONTRACTION is the most diverse (Figure 3G).

### Development of a nomogram for OS prediction

**Figure 4:**
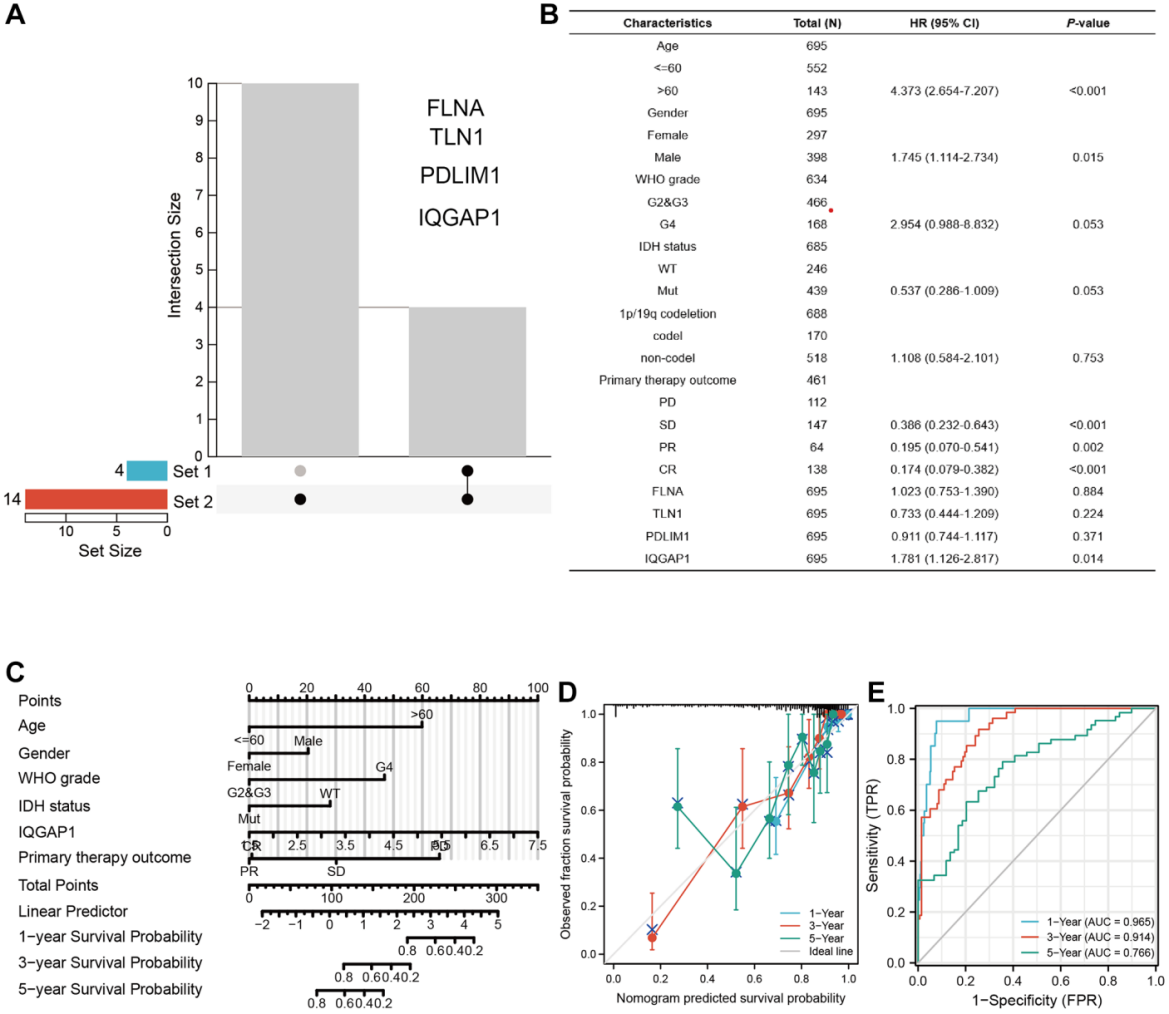
**Establishing a nomogram model in GBMLGG.** (**A**) UpSet diagram shows the common differentially expressed genes between tumor group and normal group of TCGA dataset and GEO pooled dataset. (**B**) Multivariate regression analysis was used to analyze the clinical information and differentially expressed genes of GBMLGG. (**C**) Nomogram showed the factors related to the prognosis of GBMLGG. (**D**) C-index and (**E**) ROC curve were used to calculate the reliability and accuracy of the Nomogram model.

An UpSet plot (Figure 4A) was used to screen the intersection of differentially expressed genes from the TCGA and GEO datasets. Through multivariate investigation, we screened the variables related to the operating system (Figure 4B). A list with age, gender, WHO grade, IDH status, IQGAP1, primary therapy outcome, and risk scores was made to predict OS in GBMLGG patients from the TCGA cohort (Figure 4C). The nomogram’s predicted survival probability is represented by the C-index (Figure 4D). Using the nomogram, AUC demonstrated certainty of prediction for prognosis, reaching 0.965 at one year, 0.914 at three years, and 0.766 at five years (Figure 4E).

### DNA methylation and immune cell infiltration

The CPTAC dataset uncovered that the IQGAP1 absolute protein articulation was essentially more noteworthy in essential growth tissues than in ordinary tissues (Supplementary Figure 1A). We investigated the impact of the tumor microenvironment (TME) on patient outcomes and responses to therapy, with a particular focus on tumor infiltrating immune cells (TIICs), which have a major effect on growth movement and the effectiveness of therapy (Supplementary Figure 1B). The typical degree of the IQGAP1 bunch was 4.507 ± 1.063, and the typical degree of cg17891123 bunch was 0.349 ± 0.176. Relationship examination of IQGAP1 and cg17891123: There is a negative correlation between IQGAP1 and cg17891123, with a Pearson correlation coefficient of r = −0.467 and a *P*-value of 0.001. Connection examination of IQGAP1 and cg06327621: Pearson connection coefficient r = −0.583, *P* < 0.001, showing a negative relationship somewhere in the range of IQGAP1 and cg06327621 (Supplementary Figure 1C).

### The oncogenic roles of IQGAP1 in glioma U87 cells

**Figure 5:**
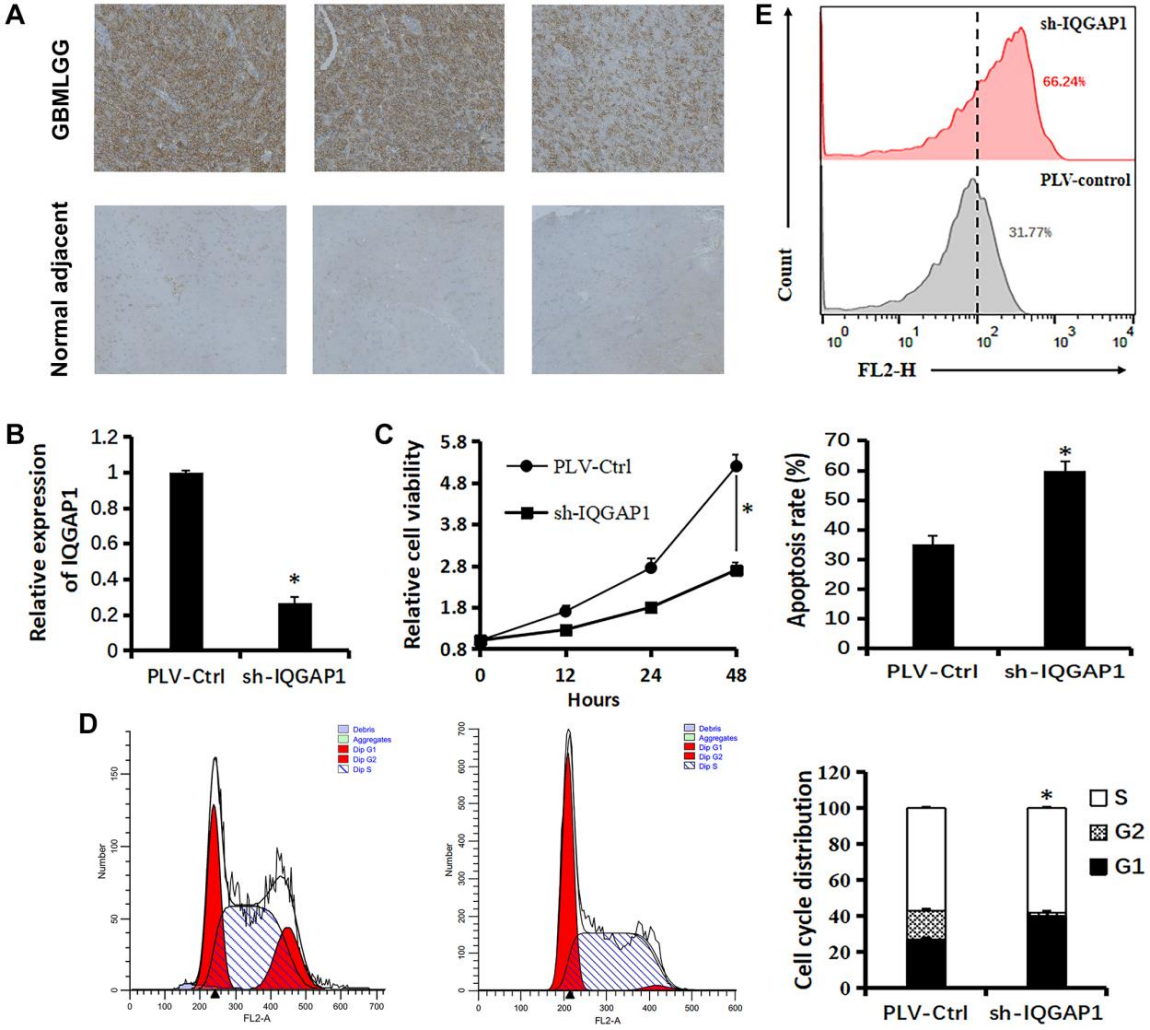
**Relationship between IQGAP1 and viability of U87 cells.** (**A**) Immunohistochemistry of IQGAP1 protein expression in GBMLGG and counterpart normal adjacent tissues. (**B**) qRT-PCR assay shows the transcriptional levels of the IQGAP1 gene with GAPDH used as the loading control in U87 cells. (**C**) Effect of sh-IQGAP1 on the proliferation of U87 cells was detected by CCK-8 assays. (**D**) The cell cycle distribution of U87 cells were measured using flow cytometry analyses. (**E**) Apoptosis of U87 cells induced by cisplatin (20 μM) were measured by flow cytometry analyses. Data are presented as the mean ± SD for three independent experiments (^*^P < 0.05).

**Figure 6:**
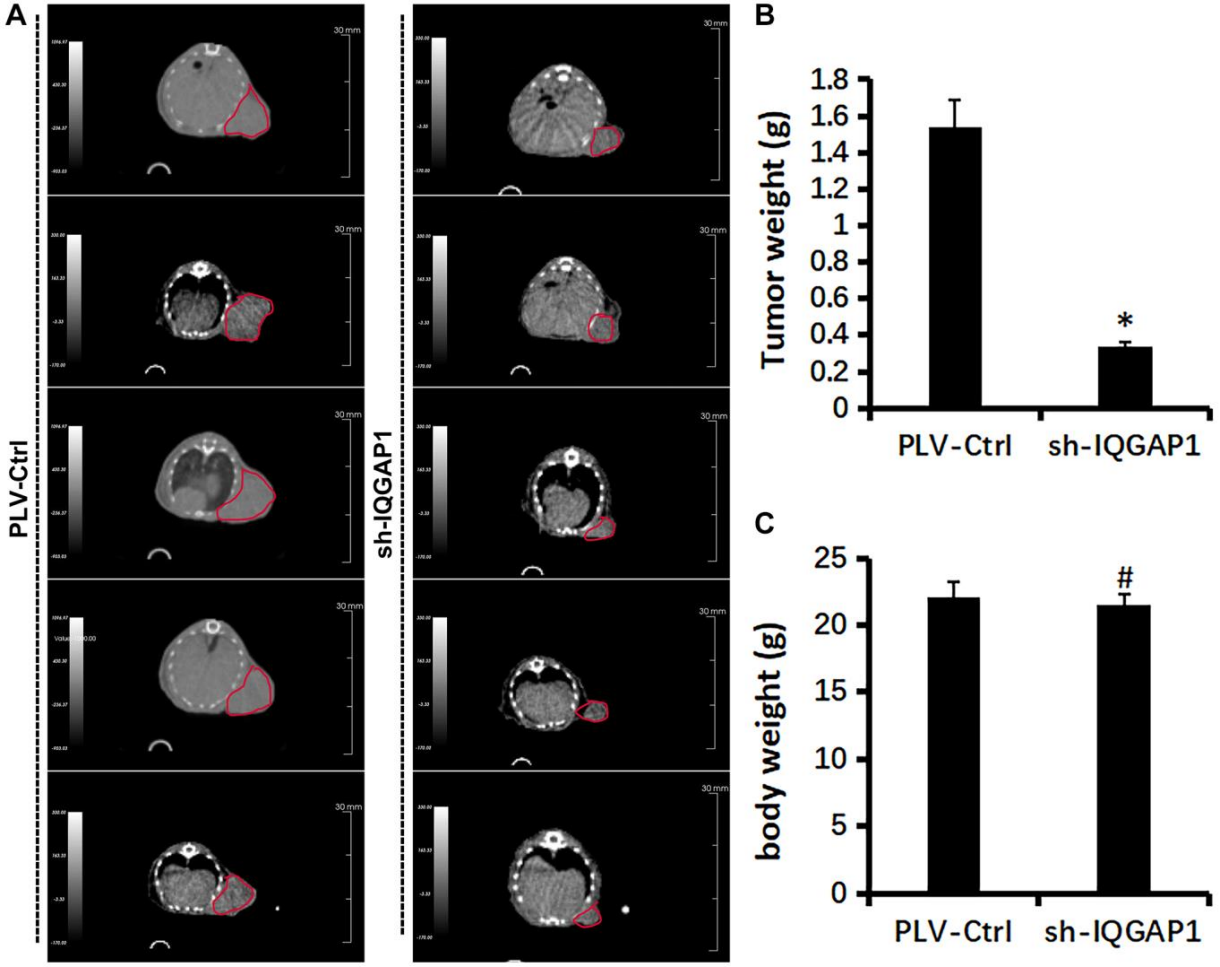
**Effect of IQGAP1 knockdown on tumor growth of human glioma U87 xenografts.** (**A**) Representative tumor images by Super Nova^®^ PET/CT at 14th day after tumor implantation. (**B**) Body weight of mice. (**C**) Tumor weight. ^*^P < 0.05, compared with the control group.

Immunohistochemistry analysis demonstrated that the protein expression level of IQGAP1 in GBMLGG were higher than those in the adjacent tissues, which indicated the oncogenic roles of IQGAP1 in glioma (Figure 5A). To further assess the roles of IQGAP1 in glioma, the shRNA vector was performed to steadily lower IQGAP1 expression in U87 cells. Utilizing quantitative continuous PCR, articulation levels were evaluated (Figure 5B). Decreased IQGAP1 expression significantly reduced cell viability (Figure 5C). The results of flow cytometry showed that sh-IQGAP1 cells contained 40% G0/G1 phase cells, while sh-Ctrl cells contained 26.5 percent G0/G1 phase cells, indicating that IQGAP1 knockdown halted the G0/G1 phase of the cell cycle (Figure 5D). Furthermore, IQGAP1 knockdown significantly enhanced the sensitivity to cisplatin treatment (Figure 5E). In addition, we used the xenografts that were injected into BALB/c mice to investigate the possibility that IQGAP1 plays a role in tumor growth (Figure 6A). When compared to the vector control, we discovered that IQGAP1 knockdown significantly inhibits tumor growth (Figure 6B), and there was no significant difference in mice’s body weight between groups (Figure 6C).

## DISCUSSION

Glioblastoma is still the most devastating brain tumor, despite clinical advancements [26]. Gene expression profiles have gained widespread acceptance as OS predictors in GBM patients ever since the inclusion of IDH mutation status in the WHO classification in 2016 [27]. Mining large amounts of genetic data has become increasingly appealing as bioinformatics and high-throughput sequencing advance. So, it’s important to know about growth administrative examples and center qualities linked to GBM guess and approve them both *in vitro* and *in vivo*.

The hallmark of cancer is altered metabolism, which presents a vulnerability in cancer treatment [28]. RCD assumes a significant role in disease metabolic treatment. Disulfidptosis is a novel metabolic-related RCD that has been identified in a recent publication [29]. According to preclinical data, GLUT inhibitors, which are employed in metabolic therapy, have the ability to induce disulfideptosis and halt the growth of cancer [28].

The development of precise prognostic indicators is crucial due to the GBMLGG’s heterogeneity. Subatomic prognostic markers are getting more and more attention because they can be used as a useful addition to the usual clinicopathological boundaries [30]. To defeat growth heterogeneity, numerous atomic markers are expected to all the more likely mirror the forecast of GBMLGG [31]. Until this point in time, the current review is quick to examine the connection between qualities engaged with disulfide bond passing and GBMLGG. 14 of the 15 genes related to disulfide breakdown that were expressed in GBMLGG samples were linked to the prognosis of GBMLGG patients. Consequently, we distinguished four qualities related to GBMLGG by consolidating the TCGA dataset and the GEO dataset. At long last, we utilized multivariate relapse examination to distinguish IQGAP1 as a center quality influencing the guess of GBMLGG. This study’s prognostic risk score model can be used to comprehensively examine the role of related genes in the prognosis of GBMLGG patients and to identify disulfide bond degradation-related therapeutic targets. The prognostic risk scoring model’s potential application value was also evaluated using a risk assessment nomogram.

This study’s core gene was therefore identified as IQGAP1. By constructing various signaling complex scaffolds, IQGAP1 aids in the progression of cancer [32]. New roles for IQGAP1 in cancer signal transduction have been discovered over the past ten years [33]. Because IQGAP1 interacts with so many different proteins, it is important to study how these proteins change over time in cancer. Because many types of cancer overexpress IQGAP1 and/or depend on signaling through IQGAP1, therapies that target IQGAP1 and its related signaling may be helpful once we understand how IQGAP1 works in cancer [34].

This study first validated the oncogenic roles of IQGAP1 in glioma using Immunohistochemistry analysis, which showed that the protein expression level of IQGAP1 in GBMLGG were higher than those in the normal adjacent tissues. The oncogenic function of IQGAP1 was further demonstrated by this study. Among them, the *in vitro* experiments were basically conducted on glioma U87 cells. Utilizing cell expansion examine and quantitative continuous PCR, we found that IQGAP1 could advance the multiplication of U87 cells. Using the experimental glioma xenograft model, we discovered that IQGAP1 aided in the *in vivo* expansion of the GBMLGG tumor. Later on, huge scope clinical preliminaries can be directed to additional review of the clinical change of IQGAP1 in advancing GBMLGG malignant growth.

## Supplementary Materials

Supplementary Figure 1
