# Supplementary figures and images for: Identification of a novel disulfideptosis-related gene signature for prognostic implication in lower-grade gliomas
Source: Aging (Albany NY). 2024 Mar 27;16(7):6054–67. doi: 10.18632/aging.205688 (PMC11042955; doi:10.18632/aging.205688)

SUPPLEMENTARY FIGURE

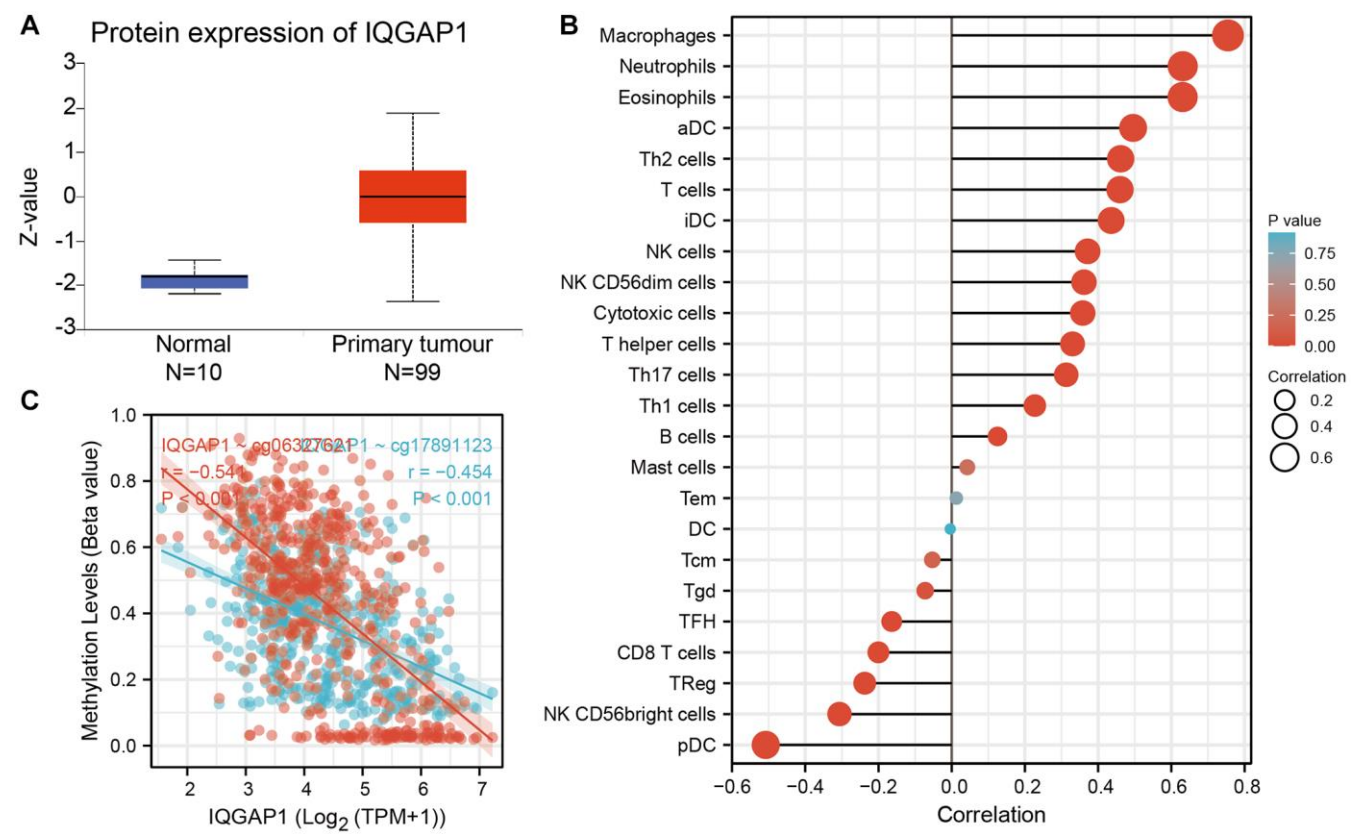

Supplement: Supplementary Figure 1 [file aging-16-205688-s001.pdf]
